# Reducing Postharvest Losses during Storage of Grain Crops to Strengthen Food Security in Developing Countries

**DOI:** 10.3390/foods6010008

**Published:** 2017-01-15

**Authors:** Deepak Kumar, Prasanta Kalita

**Affiliations:** ADM Institute for the Prevention of Postharvest Loss, University of Illinois at Urbana-Champaign, Urbana, IL 61801, USA; pkalita@illinois.edu

**Keywords:** postharvest losses, food security, grain storage, smallholders, hermetic storage

## Abstract

While fulfilling the food demand of an increasing population remains a major global concern, more than one-third of food is lost or wasted in postharvest operations. Reducing the postharvest losses, especially in developing countries, could be a sustainable solution to increase food availability, reduce pressure on natural resources, eliminate hunger and improve farmers’ livelihoods. Cereal grains are the basis of staple food in most of the developing nations, and account for the maximum postharvest losses on a calorific basis among all agricultural commodities. As much as 50%–60% cereal grains can be lost during the storage stage due only to the lack of technical inefficiency. Use of scientific storage methods can reduce these losses to as low as 1%–2%. This paper provides a comprehensive literature review of the grain postharvest losses in developing countries, the status and causes of storage losses and discusses the technological interventions to reduce these losses. The basics of hermetic storage, various technology options, and their effectiveness on several crops in different localities are discussed in detail.

## 1. Introduction

Meeting the food demand of a rapidly increasing global population is emerging as a big challenge to mankind. The population is expected to grow to 9.1 billion people by the year 2050, and about 70% extra food production will be required to feed them [1,2,3]. Most of this population rise is expected to be attributed to developing countries, several of which are already facing issues of hunger and food insecurity. Increasing urbanization, climate change and land use for non-food crop production, intensify these concerns of increasing food demands. In the last few decades, most of the countries have focused on improving their agricultural production, land use, and population control as their policies to cope with this increasing food demand. However, postharvest loss (PHL), a critical issue, does not receive the required attention and less than 5% research funding has been allocated for this issue in previous years [4,5,6,7]. Approximately one-third of the food produced (about 1.3 billion ton), worth about US $1 trillion, is lost globally during postharvest operations every year [8]. “Food loss” is defined as food that is available for human consumption but goes unconsumed [9,10]. The solutions to reduce postharvest losses require relatively modest investment and can result in high returns compared to increasing the crop production to meet the food demand.

Postharvest loss includes the food loss across the food supply chain from harvesting of crop until its consumption [9]. The losses can broadly be categorized as weight loss due to spoilage, quality loss, nutritional loss, seed viability loss, and commercial loss [11]. Magnitude of postharvest losses in the food supply chain vary greatly among different crops, areas, and economies. In developing countries, people try to make the best use of the food produced, however, a significant amount of produce is lost in postharvest operations due to a lack of knowledge, inadequate technology and/or poor storage infrastructure. On the contrary, in developed countries, food loss in the middle stages of the supply chain is relatively low due to availability of advanced technologies and efficient crop handling and storage systems. However, a large portion of food is lost at the end of the supply chain, known as food waste. “Food waste” can be defined as food discarded or alternatively the intentional non-food use of the food or due to spoilage/expiration of food [12]. In 2010, estimates suggested that about 133 billion pounds of food (31% of the total available food) was wasted at retail and consumer level in the United States. Among different agricultural commodities, the studies estimated that on a weight basis, cereal crops, roots crops, and fruit and vegetables account for about 19%, 20%, and 44% losses respectively [8,13]. On a calorific content basis, losses in cereal crops hold the largest share (53%). Cereal grains, such as wheat, rice, and maize are the most popular food crops in the world, and are the basis of staple food in most of the developing countries. Minimizing cereal losses in the supply chain could be one resource-efficient way that can help in strengthening food security, sustainably combating hunger, reducing the agricultural land needed for production, rural development, and improving farmers’ livelihoods.

Postharvest loss accounts for direct physical losses and quality losses that reduce the economic value of crop, or may make it unsuitable for human consumption. In severe cases, these losses can be up to 80% of the total production [14]. In African countries, these losses have been estimated to range between 20% and 40%, which is highly significant considering the low agricultural productivity in several regions of Africa [15]. According to the World Bank report, sub-Saharan Africa (SSA) alone loses food grains worth about USD 4 billion every year [16]. These losses play a critical role in influencing the life of millions of smallholder farmers by impacting the available food volumes and trade-in values of the commodities. In addition to economic and social implications, postharvest losses also impact the environment, as the land, water and energy (agricultural inputs) used to produce the lost food are also wasted along with the food. Unutilized food also results in extra CO_2_ emissions, eventually affecting the environment. A report from the Food and Agriculture Organization of the United Nations (FAO) using the life cycle perspective, estimated about 3.3 Gtonnes of CO_2_ equivalent emissions due to food that was produced but not eaten, without even considering the land use change [17]. The blue water footprints (water use during life cycle of food) for the wasted food globally was estimated to be about 250 km^3^ [14,17]. Similarly, the land used to grow the food is another valuable resource that goes to waste due to these losses. A study conducted on rice postharvest losses in Nigeria estimated that the lost paddy accounted for 19% of the total cultivated area [18]. On the global scale, about 1.4 billion hectares of land was wasted by growing food that was not consumed in the year 2007, an area larger than Canada and China [19]. 

Considering the criticality of PHL reduction in enhancing the food security, it becomes very important to know the pattern and scale of these losses across the world, especially in developing countries, and identify its causes and possible solutions. Although losses occur at each stage of the supply chain from production to consumer level, storage losses are considered most critical in developing countries. This paper provides a comprehensive review and discussion on the status of storage losses of major cereal crops, major factors that lead to these losses and possible solutions. Technology interventions play a critical role in addressing the issue of PHL, and several efforts have been made to develop and disseminate these technologies for smallholders in developing countries. However, there is a lack of compiled evidence-based information on the effectiveness of these technologies for various crops. This paper discusses in detail the technology interventions, especially the use and effectiveness of hermetic storage in reducing storage losses particularly for smallholders in developing countries.

## 2. Grain Supply Chain

During the crop transition from farm to consumer, it has to undergo several operations such as harvesting, threshing, cleaning, drying, storage, processing and transportation. During this movement, crop is lost due to several factors such as improper handling, inefficient processing facilities, biodegradation due to microorganisms and insects, etc. It is important to understand the supply chain and identify factors at various stages that cause food losses. The section below will discuss the various stages in grain supply chain and type of losses occurring at each stage.

### 2.1. Harvesting

Harvesting is considered as the first step in the grain supply chain and is a critical operation in deciding the overall crop quality. In the developing countries, crop harvesting is performed mainly manually using hand cutting tools such as sickle, knife, scythe, cutters. Almost all of the crop is harvested using combine harvesters in the developed countries.

Harvesting timing and method (mechanical vs. manual) are two critical factors dictating the losses during the harvesting operations. A large amount of losses occurs before or during the harvesting operations, if it is not performed at adequate crop maturity and moisture content. Too early harvesting of crop at high moisture content increases the drying cost, making it susceptible to mold growth, insect infestation, and resulting in a high amount of broken grains and low milling yields [20]. However, leaving the matured crop un-harvested results in high shattering losses, exposure to birds and rodents attack, and losses due to natural calamities (rain, hailstorms etc.) [21]. Most of the harvesting is performed manually in the developing countries, which is a highly labor intensive and slow process. During peak harvesting season, even the countries such as India and Bangladesh encounter labor shortages, which results in delays in the harvesting and subsequently large losses. According to a study conducted in Punjab, India, due to high shattering losses, the wheat harvesting losses were found increased by about 67% (2.5% from 1.5%) by delay in harvesting [22]. Another postharvest loss study in India estimated a 10.3% increase (1.74% to 1.92%) in paddy harvesting losses due to delayed harvesting because of a lack in adequate harvesting equipment [23]. The recommended optimum moisture content during harvesting of various crops is listed in Table 1. 

### 2.2. Threshing and Cleaning

The purpose of the threshing process is to detach the grain from the panicles. The process is achieved through rubbing, stripping, or impact action, or using a combination of these actions. The operation can be performed manually (trampling, beating), using animal power, or mechanical threshers. Manual threshing is the most common practice in the developing countries. Grain spillage, incomplete separation of the grain from chaff, grain breakage due of excessive striking, are some of the major reasons for losses during the threshing process [20,25]. Delay in threshing after harvesting of crop results in significant quantity and quality loss, as the crop is exposed to atmosphere, and is susceptible to rodents, birds, and insect attack [26]. As in the case of harvesting, lack of mechanization is the major reason for this delay that causes significant losses. High moisture accumulations in the crop lying in the field may even lead to start mold growth in the field. 

The cleaning process is performed after the threshing to separate whole grains from broken grains and other foreign materials, such as straw, stones, sand, chaff, and weed seed. Winnowing is the most common method used for cleaning in the developing countries. Screening/sifting is another common method of cleaning, which can be performed either manually or mechanically. Inadequate cleaned grains can increase the insect infestation and mold growth during storage, add unwanted taste and color, and can damage the processing equipment. A large amount of grains are lost as spillage during this operation, and grain losses during winnowing can be as high as 4% of the total production [27].

### 2.3. Drying

As apparent from the Table 1, the grains are usually harvested at high moisture content to minimize the shattering losses in the field. However, the safe moisture content for long-term storage of most of the crops is considered below 13% [21]. Even for the short-term storage (less than 6 months), the moisture should be less than 15% for most of the crops. Inadequate drying can result in mold growth and significantly high losses during storage and milling. Therefore, drying is a critical step after harvesting to maintain the crop quality, minimize storage losses and reduce transportation cost. 

Drying can be performed naturally (sun or shade drying) or using mechanical dryers. Natural drying or sun drying is the traditional and economical practice for drying the harvested crop, and is the most popular method in developing countries. Sometimes, whole crop without threshing is left in the field only for drying. For example, after wheat harvesting, stacks are made of 10–15 bundles of tied crop, and left in the field for drying. Sun drying is weather dependent, requires high labor, is slow, and causes large losses. Grains lying in the open for sun drying are eaten by birds and insects, and also get contaminated due to mixing of stones, dust, and other foreign materials. Unseasonal rains or cloudier weather may restrict the proper drying, and the crop is stored at high moisture, which leads to high losses due to mold growth. About 3.5% and 4.5% losses were reported during maize drying on raised platforms in Zambia and Zimbabwe respectively [15,28]. Some farmers use mats or plastic sheets for spreading the grains, which reduces the contamination with dust and makes the collection of grains easy. Mechanical drying addresses some of the limitations of natural drying, and offers advantages, such as reduction in handling losses, better control over the hot air temperature, and space utilization. However, they suffer with the limitations of high initial and maintenance cost, adequate size availability, and lack of knowledge to operate these dryers, especially with smallholders. Due to these limitations, these dryers are rarely used by smallholders in the developing countries [26].

### 2.4. Storage

Storage plays a vital role in the food supply chain, and several studies reported that maximum losses happen during this operation [9,29,30]. In most of the places, crops are grown seasonally and after harvesting, grains are stored for short or long periods as food reserves, and as seeds for next season. Studies report that in developing countries such as India, about 50%–60% of the grains are stored in the traditional structures (e.g., Kanaja, Kothi, Sanduka, earthen pots, Gummi and Kacheri) at the household and farm level for self-consumption and seed [22]. The indigenous storage structures are made of locally available materials (grass, wood, mud etc.) without any scientific design, and cannot guarantee to protect crops against pests for a long time. Costa [31] estimated losses as high as 59.48% in maize grains after storing them for 90 days in the traditional storage structures (Granary/Polypropylene bags). The causes of losses during grain storage will be discussed in detail in a later section.

### 2.5. Transportation

Transportation is an important operation of the grain value chain, as commodities need to be moved from one step to another, such as field to processing facilities, field to storage facilities, and processing facilities to market. The lack of adequate transportation infrastructure results in damage of food products through bruising and losses due to spillage. Transportation loses are relatively very low in the developed countries due to better road infrastructure and engineered facilities on the field and processing facilities to load and unload the vehicles rapidly with very little or no damage. At the field level, most of the crop is transported in bullock carts or open trollies in South Asian countries. Grains for self-usage are usually transported in bags from field storage to processing facilities in bullock carts, bicycles, small motor vehicles, or open trucks. Poor road infrastructure along with these improper and poorly maintained modes of transportation results in large spillage and high contamination. Multiple movements of crop is another major reason for high transportation losses. In countries such as India and Pakistan, sometimes bagged wheat is loaded and unloaded from vehicles up to ten times before it is milled [21]. During each movement some grains are lost as spillage. Unlike efficient bulk handling systems in developed countries, loading and unloading of grains from wagons, trucks, and rails at processing facilities is performed mostly manually in the developing nations, and results in high spillage. Low quality Jute bags are used commonly during transportation and even storage, which results in high spillage rates due to leakage from the sacks. Large quantities (usually 100 kg of grains) in each bag, and hooks used to lift these bags cause tear in these bags and results in high spillage [21]. Even the trucks used in the developing countries are not totally suitable to transport cereals and oil seed crops. Alavi et al. [26] reported 2%–10% losses during handling and transportation of rice in Southeast Asia.

### 2.6. Milling

The milling or processing operations vary for different grains. In the case of rice, the purposes of milling are to remove the husk and bran layers of paddy to provide cleaned and whole white rice kernels for human consumption. The operation can be performed manually or using milling machines. Traditionally, in rural areas, milling is performed manually by repeated pounding. Milling yields are highly dependent on the milling method, skills of the operator, and crop conditions before the milling process. Milling of paddy containing foreign materials results in a high amount of cracked and broken kernels and can also damage machines. Inadequately maintained milling machines result in a high amount of broken kernels and low milling yields. Alavi et al. [26] reported that milling losses are highest among the losses during postharvest operations of rice in five Southeast countries: China, Thailand, Indonesia, the Philippines, and Vietnam. Milling yields of rice in all these five countries were reported well below the theoretical yield of 71%–73%. The yields from village level small mills were as low as 57% due to small scale, poor calibration, and lack of maintenance. High moisture and an inadequately cleaned paddy aggravate the situation and reduce yields.

**Figure 1:**
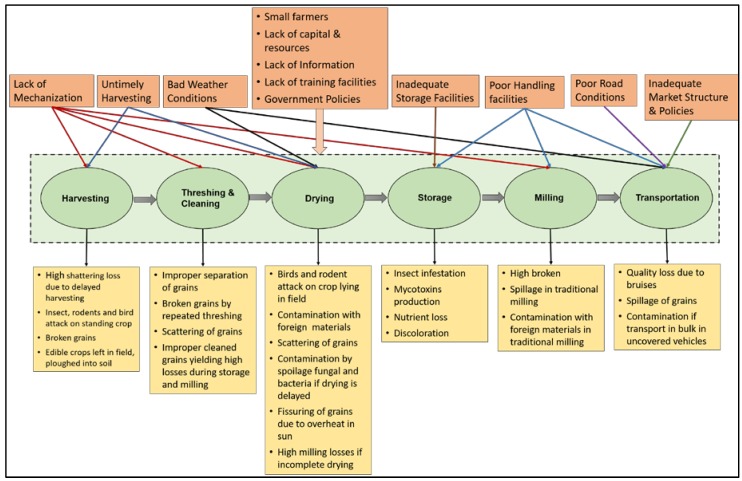
Various factors and types of losses during the supply chain of cereal crops in developing countries.

Figure 1 summarizes various losses that occur during the supply chain of cereal crops and major factors responsible for those losses in the developing countries.

## 3. Postharvest Losses of Cereal Crops in Developing Countries

Rice, wheat, and maize are major cereal grains in most of the developing countries. In countries such as Bangladesh, rice accounts for more than 90% of food produced and about 70% of calories intake [32]. In West Africa, Nigeria is currently the largest producer of rice with an annual production of about 3.3 million tonnes [18]. In spite of the large production and huge rice imports every year, a large number of people are undernourished in Nigeria. Similarly, Bangladesh is the fourth largest producer of rice worldwide, however, it is still food deficient and imports more than one million tons of rice every year. Saving the cereal crop lost during postharvest operations can help in meeting the food demand and reduce the load on the economy. A report from the World Bank, estimated 7%–10% of grain loss in postharvest operations at field level, and 4%–5% loss at the market and distribution stage in India for the year 1999 [25,33,34]. Estimates also suggest that these, approximately 12 to 16 million metric tons of grains wasted each year, could meet the food demand of about one-third of India’s poor population [35]. However, despite the criticality of the issue, availability of consistent and reliable postharvest loss data is still a challenge. Very few loss assessment studies have been conducted in important developing economies such as India, China, and Brazil. After the FAO report in 2010, various institutes are making efforts to conduct comprehensive household surveys, interviews, and field measurements to determine the actual status of losses along food supply chains in various countries. This section discusses the status of losses in major staple crops based on the available literature data. 

### 3.1. Rice

**Figure 2:**
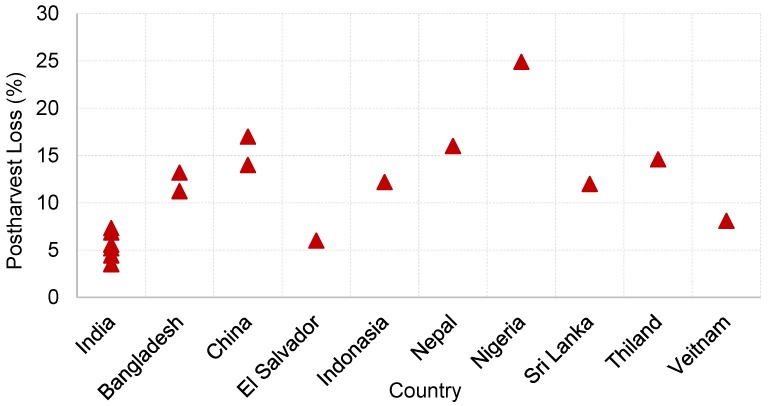
Postharvest losses in the rice value chain in various countries (in the case of a range of losses, an average of losses was used).

Being a high energy calorie food, rice accounts for one-fifth of the global calorie supply. The scale of postharvest losses in grain supply chains varies significantly, depending on the economy, agricultural conditions and practices, and climatic conditions of the region. An example of such variation of losses in the rice supply chain in different countries is illustrated in Figure 2. The rice losses have been reported as low as 3.51% in India to as high as 24.9% in Nigeria (Figure 2). Based on the 24.9% loss, the value of the total grain loss during the rice supply chain in Nigeria was estimated as 56.7 billion Nigerian Naira (NGN). According to Bala et al. [29], the losses in the rice value chain from producer to retailer were estimated as 10.74% to 11.71% (10.74% for Aman, 11.71% for Boro, and 11.59% for Aus) in Bangladesh. Most of the losses (85.28%–87.77% of the total) happened in the farm level operations, with storage losses (33.92%–40.99% of farm level losses) being the main contributor. Alavi et al. [26] compiled data on postharvest losses in rice value chains from different studies conducted by the FAO and reported 10%–37% losses in rice in Southeast Asia. In China, the losses were estimated in the range of 8%–26%.

### 3.2. Wheat

Wheat is another major staple food of several countries in Europe, Asia, and North America. Similar to rice, significant losses happen during postharvest processing of wheat in developing countries. According to data compiled (before the year 1978) by US National Academy of Sciences, wheat losses in Sudan and Zimbabwe were estimated 6%–19% and 10% respectively [36]. Bala et al. [29] reported that the storage losses were maximum (41.7% of the total) among all the postharvest operation losses for wheat in Bangladesh, even considering the fact that the storage period of wheat is relatively small. Basavarja et al. [33] conducted a study to estimate losses in postharvest operations of wheat in the state of Karnataka, India. The estimations were based on a comprehensive survey from 100 farmers, 20 wholesalers, 20 processors and 20 retailers from the major producer district of each crop in the Karnataka state. The overall losses in the wheat supply chain from harvesting to retailer were estimated as 4.32%. Field level operations contributed 75.9% among the total postharvest losses (Table 2). The losses were maximum during storage operations due to a lack of availability, poor structures, presence of rodents, and improper drainage. 

### 3.3. Maize

Maize is an important part of staple food in Sub-Saharan Africa (SSA) and a major source (~36%) of daily calories intake. Pantenius [7] estimated 0.2%–11.8% weight loss due to insect infestation in maize after 6 months of storage in traditional granaries in Togo. Inter-American Institute for Cooperation on Agriculture (IICA) conducted a survey to estimate the postharvest losses in Latin America and Caribbean countries. The losses values estimated in various regions have been presented in the table below (Table 3). In almost all regions, most of the losses were observed occurring at the small and medium-scale farms due to a lack of adequate harvesting, drying, and storage technologies, along with a lack of information about the good agricultural practices. In Guatemala, due to a lack in storage structures along with the region’s high humidity, storage losses were estimated between 40% and 45% [40]. Insect infestation was found as the major reason of storage losses in most of the cases. Kaminski and Christiansen [42] conducted a study to estimate the postharvest losses in maize crop in three SSA countries (Uganda, Tanzania, and Malawi) through comprehensive household surveys. The losses from the farm level activities were estimated in the range of 1.4% to 5.9%. Insects and pests were reported as the major reason of losses in maize during storage. Alavi et al. [26] reported an average of 23% losses in the maize value chain in ASEAN (the Association of Southeast Asian Nations) countries, with maximum losses happening during field drying (9%). Most of the maize is dried along the sides of road, especially in the Philippines. In Vietnam, major losses occur due to rodent attack and fungal disease during maize storage.

## 4. Storage Losses in Developing Countries

As discussed in the above section, the maximum amount of losses occurs during the storage of crops due to a lack of adequate infrastructure. Storage losses can be classified in two categories: direct losses, due to physical loss of commodities; and indirect losses, due to loss in quality and nutrition. It is important to consider both damage and losses by the insects during storage instead of just weight loss. “Damage” can refer to physical evidence of deterioration, for example, holes in the grains. It mainly affects the quality of grains. “Loss”, on the other side, is the total disappearance of the food, which can be measured quantitatively [43]. The loss in quality results in value loss of the product, and sometimes leads to total rejection also. The rejection rate depends upon the individual’s economic status and cultural background. For example, a subsistence farmer may consume the damaged food to some extent, whereas, affluent customers may reject even slightly damaged food. Some loss happens in the form of spillage from leaky sacks which can be identified when the store is emptied and the spilt grain remains on the floor.

The storage losses are affected by several factors, which can be classified into two main categories: biotic factors (insect, pest, rodents, fungi) and abiotic factors (temperature, humidity, rain) [32]. Moisture content and temperature are the most crucial factors affecting the storage life. Most of the storage molds grow rapidly at temperatures of 20–40 °C and relative humidity of more than 70% [32]. Low moisture keeps the relative humidity levels below 70% and limits the mold growth. In the traditional storage structure, temperature fluctuations due to weather changes cause moisture accumulation either at the top or bottom of the grains’ bulk depending on the direction of air convection. This can be avoided by minimizing the temperature difference of inside and outside the storage structure. Grains should be dried to about 13% of the moisture content before storage to minimize the losses. At moisture contents of 16% or higher, the safe storage period of rice is only a few weeks [32]. Quality of grains before storage is another critical factor affecting the storage losses. Mechanical damage during harvesting and threshing can result in bruised areas on grains, which may serve as centers for infection and cause deterioration [25]. The criticality of a factor depends upon the storage conditions. 

In most of the developing countries, especially in Africa and South Asia, grains are generally stored as bulk or bags in simple granaries constructed from locally available materials (straw, bamboo, mud, bricks). Mud bins and pots, bokharies (straw structure), kothis, and plastic containers are common storage structures in Asia [21]. Gunny bags and Plastic/Polythene bags are commonly used for the short duration storage, and Dole, Berh, Gola, Motka, Steel/Plastic drums are used for the long duration storage. Various types of granaries are used in African countries. Ebli-va, Kedelin, in-house smoked storage are some of the common maize storage structures in Togo [7]. In the in-house smoked storage methods, maize is stored within the dwelling in space between the ceiling and roof over the cooking spot to receive the heat. In West Africa, grains are commonly stored in the home or field in jute or polypropylene bags, raised platforms, conical structures, and baskets [15,44]. In East and Southern Africa, farmers use cow dung ash in small bags, wood cribs, pits, iron drums enclosed with mud, and metal bins for storing the grains [15,45]. “Nkokwe” is one of the most commonly used storage structure used in Malawi and Kenya. This is a kind of cylindrical basket made up of interwoven split bamboo and covered with a conical shaped roof of grass. The structure is raised off the ground on stilts [36]. Most of these structures are not scientifically designed and are made from locally available materials, and cause damage to stored grains due to biological, environmental and other factors. 

### 4.1. Insect Infestation

Among all the biotic factors, insect pests are considered most important and cause huge losses in the grains (30%–40%) [15,43,46]. Some studies in Ghana reported that the maize losses due to insect infestation could be up to 50%, if all quantity losses, quality losses, and income loss due to early sale are considered in the estimation [43]. According to an economic model by Compton et al. [47], each percent of insect infestation results in 0.6%–1% depreciation in the value of maize [47,48]. From field studies in Togo, Pantenius [7] observed that insects and pests were responsible for 80%–90% of storage losses in grains. *Callosobruchus maculatus* (F.) alone, a common pulse weevil has been found responsible for up to 24% losses in stored pulses in Nigeria [46]. Losses due to insects in stored maize have been reported from 12% to 44% in the western highlands of Cameroon [46]. The maize weevil (*Sitophilus zeamais*), and larger grain borer (LGB) (*Prostephanus truncatus*) are the major pests in the maize. About 23% losses were observed in maize grains stored for six months, mainly due to infestation of maize weevil and LGB in Benin [49,50]. LGB originated in Central America and was accidently introduced in Africa in late 1970s [49]. Nowadays, it is found in most parts of Africa and is considered the most threatening pest, as it causes extensive damage in a very short time [43,51,52]. The sporadic nature of LGB makes even its control difficult: it does not infest all stores of the same area, and its reoccurrence in each year is not guaranteed. At farm level storage, more than 30% of weight loss have been observed in maize due to these pests [43,52]. Some studies on maize losses in Ghana estimated about 5% to 10% loss in market value due to infestation by only *Sitophuilus* spp., and 15% to 45% market value loss due to damage by LGB. Overall, these losses were equivalent to about 5% of the average household income in that area [43,53,54]. Abass et al. [15] reported that after six months of maize storage, LGB was responsible for more than half (56.7%) of the storage losses, followed by losses due to grain weevil and lesser grain borer. Patel et al. (1993) observed about 25% losses by *R. dominica* in wheat stored for 3 months under laboratory conditions [55]. 

### 4.2. Mycotoxins

Mycotoxin contamination is another big challenge, especially in the case of maize, which makes the food unsuitable for human consumption or animal feed. A large amount (25%–40%) of cereal grains are contaminated by the mycotoxins produced by storage fungi world-wide [56]. Molds and mycotoxins cause dry matter as well as quality loss, and are a hazard in the food value chain [57]. Aflatoxins, Fumonisins, Deoxynivalenol, and Ochratoxin are the most common and important mycotoxins, especially in maize [58,59,60]. Aflatoxins, produced as secondary metabolite by two fungi species *Aspergillus flavus* and *A. parasiticus*, are considered the most dangerous group of mycotoxins, as they increase the risk of liver cancer and affect growth in young children [59,61]. Because of food contamination, about 4.5 billion people are exposed to aflatoxins in developing countries [56,62]. High concentrations of aflatoxin can lead to aflatoxicosis, which can cause severe illness and even death [63]. *Penicillium verrucosum* (ochratoxin), a major mycotoxigenic mold is commonly found in damp cool climates (e.g., Northern Europe) and *Aspergillus flavus* is mostly observed in temperate and tropical conditions [57]. 

Mold during storage damages the grains as well as reducing grain germination. It also deteriorates the grain quality due to the must odour, increased fatty acid content, and reduced starch and sugar contents. Lipid peroxidation is another phenomenon that causes food deterioration and alters the taste and aroma, and may cause undesirable effects on human health [56]. High oil content varieties of oil seeds demand particular attention during storage, as the high level of moisture degrades the vegetable oil and produces high fatty acids, which sometimes also results in self-heating [14]. At farm level storage in developing countries, even rodents can damage a large portion of crop, whereas, fungi can be a major reason for spoilage at high relative humidity storage. Use of scientific storage structures and proper handling of grains can reduce storage losses to less than 1% [31,64]. 

Losses can be minimized by physically avoiding the entry of insects and rodents, and maintaining the environmental conditions that avoid growth of microorganisms. The knowledge of control points during harvesting and drying before storage can help in reducing losses during the storage of cereals. Taking the timely preventive actions for biotic and abiotic factors can be very effective in reducing the losses during storage.

## 5. Interventions to Reduce Storage Losses for Smallholders

Although a huge challenge, storage losses can be mitigated by use of efficient storage technology, upgrading infrastructure and storage practices. World Food Programme (WFP) with the help of the government and non-governmental organizations (NGOs) performed an Action Research Trial in Uganda and Burkina Faso to demonstrate the impact of improved postharvest management practices and using new storage technologies on the crop loss after harvesting [31]. The results concluded that irrespective of crop or storage periods, use of improved practices and new technologies resulted in about a 98% reduction in food loss [31]. It was also observed that losses in the traditional storage structures were much higher than those reported in the literature, because the storage period was longer than that commonly used by farmers in these countries. It is important to understand their usefulness, technical efficacy, and limitations to promote their adaptability among the consumers. This section of the paper will discuss various practices and technology interventions that can help in reducing storage losses for smallholders in developing nations. Other than saving losses, the availability of low cost and effective storage structures can motivate farmers to store their grains and obtain high prices instead of selling right after harvesting when there is an abundant supply of grains.

### 5.1. Chemical Fumigation

Synthetic insecticides are used in several countries and play an important role in controlling the pests and reducing losses during storage of grains. Methyl bromide (MB) and phosphine are the most commonly used chemicals in developing countries [65]. If the maize grains are sufficiently dry (moisture content less than 13%), use of phostoxin can control the LGB infestation in maize grains. However, phostoxin can be applied only by the licensed technicians in most parts of Africa, and farmers are allowed to use a mixture of pirimiphos-methyl (Actellic) and permethrin, commercially sold as Actellic Super [66]. Shelling the grains and storing them in polypropylene bags after proper application of Actellic Super can effectively avoid the pest infestation for a few months of storage [49,66]. This practice has been widely adopted by farmers in African countries, especially Kenya. More than 93% of farmers reported this as the most common method used for controlling pest during storage in Tanzania [49].

Irrespective of their effectiveness, the synthetic insecticides suffer from limitations such as high costs, development of genetic resistance in the treated pests, health hazards due to toxic residues, and environmental contamination [46,65]. Residuals from synthetic fumigants could cause considerable loss of seed viability [67]. Due to long use of phosphine, some insects have gained resistance to chemical fumigation in some countries [68,69]. Use of these chemical fumigation methods is even challenging in the traditional storage structures used in the developing countries, as most of them are open to reinfestation. Another challenge with these chemicals is knowledge and training to apply these pesticides at the correct time and at the correct dose. The delayed treatment, adulterated chemicals, and incorrect dosage can reduce the efficacy of the treatment and result in high storage losses.

### 5.2. Natural Insecticides

Several plant species and their extracts have been found with natural pesticide ability and are used very commonly as a traditional practice to protect the grains from insects in several African and Asian countries. Plant based chemicals and products would be biodegradable, environment friendly, and relatively safe for human health. The leaves and oil extract from leaves of *Chenopodium ambrosioides Linn.* (Chenopodiaceae) has been found to be very effective in controlling the damage of cereal grains by the insects during storage, in several studies. The plant is a branched herb and widely available in India [56]. Its natural pesticide abilities have been highlighted and investigated in several studies. Kumar et al. (2007) investigated the effectiveness of essential oil from wormseed against the fungal deterioration in stored wheat. The samples were analyzed for fungi after 12 months of storage at laboratory conditions. The oil was found to be significantly effective in controlling the *A. flavus* fungi in both inoculated (91.17% protection) and uninoculated (99.42% protection) wheat samples. The efficacy of oil was compared with synthetic fungicides (benzimidazole (Benomyl), diphenylamine (DPA), phenylmercuric acetate (Ceresan) and zinc dimethyl dithiocarbamate (Ziram)), and the oil was found to be relatively more effective, with minimum inhibitory concentration (MIC) (concentration at which the oil shows absolute fungitoxicity) lower than those of synthetic ones. Tapondjou et al. (2002) investigated the effectiveness of using leaves and extracted essential oil from wormseed (*Chenopodium ambrosioides* L.) against six common species of grain beetles in the western highlands of Cameroon: *Sitophilus zeamais* (maize weevil)., *S. granarius* (L.) (granary weevil), *Callosobruchus chinensis* (L.), *C. maculatus*, *Acanthocelides obtectus* (all Bruchidae), and *Prostephanus truncatus* (bruchids) (larger grain borer). The ground leaves were mixed with grains at the concentration of 0.05%–0.8% (*w*/*w*) for bruchids, and 0.8%–6.4% (*w*/*w*) for weevils and borers. The grains tested were maize for *S. zeamais* and *P. truncatus*, whole wheat for *S. granarius*, green peas for *C. chinensis*, mung bean for *C. maculatus* and white bean for *A. obtectus*. The oil effectiveness was checked on the filter paper discs by treating a Whatman No. 1 filter paper with the oil diluted in acetone at different concentrations of oil (0 to 1.6 µL/cm^2^). The mortality rate was found to be relatively high for bruchids (100% for *C. chinensis* in 48 h) compared to that in the case of weevils and borers. The essential oil was observed to be more toxic than using leaves and resulted in high mortality. Other than mortality, the ground leaves were effective in inhibiting the F1 progeny production and adult emergence of the insects. Shaaya et al. [65] tested four edible oils: Pure soybean oil, pure and crude cottonseed oils, crude rice bran oil and crude palm kernel oil, as fumigants against common insects in beans and wheat. Crude palm kernel and crude rice bran oils were found to very effectively control *C. maculatus* in chickpea for the initial 4–5 months. Even at the end of 15 months, the insects in the fumigated samples were only 10% of those in the control sample. Similarly, crude cotton and soya bean oils were found to be effective against *S. oryzae* in wheat for the initial 4–5 months, and were effective later at high concentrations (10 g/kg). Only crude cotton oil was found to be significantly effective in controlling *S. zeamais* in maize, and that too mainly during the initial 4 months of storage period. After 8 months of storage, the oil was partially effective and had 20%–40% of the amount of insects of those in untreated maize samples. The major issue with these plant materials is that oil yields are low and might be expensive to use on a commercial scale, however, some plant leaves can be used as natural insecticides by smallholders. 

### 5.3. Hermetic Storage

Hermetic storage (HS), also known called as “sealed storage” or “airtight storage”, is gaining popularity as a storage method for cereal, pulses, coffee, and cocoa beans in developing countries, due to its effectiveness and avoidance of the use of chemicals and pesticides. The method creates an automatic modified atmosphere of high carbon dioxide concentration using sealed waterproof bags or structures. As the structures are airtight, the biotic portion of the grains (insects and aerobic microorganisms) creates a self-inhibitory atmosphere over time by increasing carbon dioxide concentration (oxygen decreases) due to its respiration metabolism. Some studies have reported that the aflatoxin production ability of *Aspergillus flavus* is also reduced at high concentrations of CO_2_ [63,70]. Hermetic storage has been observed to be very effective in avoiding the losses (storage losses less than 1%) during long distance (international) shipments also [69]. Ease of installation, elimination of pesticide use, favorable costs, and modest infrastructure requirements are some of the additional advantages that make the hermetic storage options attractive [71].

**Figure 3:**
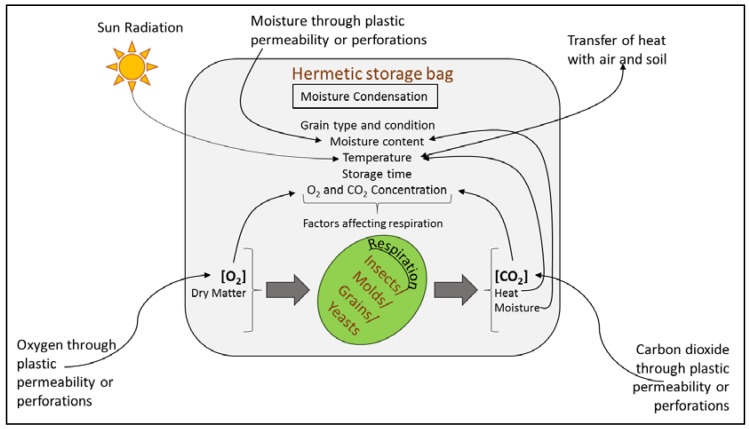
Illustration of the factors affecting the grain and microorganism respiration in the hermetic storage (Adapted from Cardoso et al. [72]).

The relationship of the main factors affecting the respiration of grain and microorganisms in hermetic storage has been described in the Figure 3 [72]. The CO_2_ concentration inside the bags is usually used as an indicator of the biological activity of grains. [72,73]. Permeability of the bag and the gas partial pressure effect the movement of gases (O_2_ and CO_2_) in and out, whereas the concentration of these gases inside the bag depends on the balance between these exchanges and the respiration of the biotic portion of grains. Higher initial moisture content tends to increase the CO_2_ concentration because of the increased respiration, however, the change was not found to be significant [72].

Another factor affecting the respiration rate is grain temperature. It has been observed in experimental studies that the temperature inside the bags follows the ambient temperature trend, and for every 10-degree increase in temperature, the CO_2_ concentration increases by about 1.5% [72]. World Food Programme (WFP) in their Action Research Trial in Uganda and Burkina Faso, found out that if properly sealed, the hermetic storage units were themselves very efficient in killing the pests and insects without any use of phosphine fumigation [31]. Various hermetic storage options, such as Metallic silos, Purdue Improved Cowpea Storage (PICS) bags, SuperGrain bags, etc., have been developed and widely promoted in the last few years. These bags are being considered practical and cost-effective storage technology, and are becoming very popular in several countries [74]. 

A metal silo is a strong hermetically sealed structure (mostly cylindrical), built using a galvanized steel sheet, and has been found to be very effective for storing grains for long periods of time and avoiding insects and rodents [75,76]. In some locations, the siloes are made of painted aluminium sheeting which helps prevent corrosion and improves their appearance [76]. It is considered to be one of the key technologies which will be helpful in reducing postharvest losses and improving food security of smallholder farmers. PICS or Purdue Improved Crop Storage bags, originally developed for storage of cowpea, involve triple bagging the grains in hermetic conditions, and is widely used by farmers in sub-Saharan Africa. The grains are stored in double layer thick (80 µm) high density polyethylene (HDPE) bags and is held in a third woven nylon bag. After filling with the grains, the bags are sealed airtight. This will cut off the oxygen to the weevils and hinder their metabolic pathways preventing them from producing water, and killing them by desiccation (Murdock et al., 2012). More than 3 million PICS bags were sold in West and Central Africa during 2007–2013. SuperGrain, commercialized by GrainPro Inc. is another widely used water resistant and hermetic storage option. These bags are made up of a single thick layer of high density polypropylene with a thickness of about 78µm, and used as liner along with normal woven polypropylene bags [59]. ZeroFly bags are a product of Vestergaard, Switzerland. These are insecticide infused woven polypropylene bags designed to prevent damaging pest infestations. The bag is made with pyrethroid incorporated into polypropylene yarns. 

**Figure 4:**
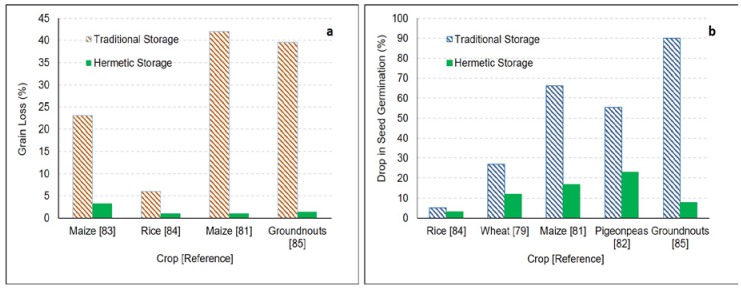
Amount of losses (**a**) weight loss; (**b**) seed germination losses, for various grains due to natural or artificial insect infestation during storage in traditional storage vs. hermetic storage (in the case of a range of losses, an average of the losses was used).

Baoua et al. [77] conducted a study to compare the performance of SuperGrain bags and PICS bags, available in the West African market, to control pest infestation over the 4 months of storage. The change in temperature and relative humidity in both the bags were similar over time. The infestation level of *C. maculatus* eggs on the seeds after four months was found to be lower in SuperGrain bags (18.5 eggs per 100 seeds) in comparison to PICS bag (26.1 eggs per 100 seeds). Grain damage was observed relatively lower (18.5 grains with holes per 100 tested grains) for the PICS bag compared to that in the SuperGrain bags (29.5 grains with holes per 100 tested grains). Somavat et al. [78] compared the effectiveness of hermetic bin bags (GrainSafe IIITM, GrainPro Inc., Concord, MA, USA), metallic bins and gunny bags for storage of wheat under ambient conditions in India. There was no insect infestation found in clean grains stored in hermetic bags after 9 months of storage. For the artificially infested grains, bored grain percentage remained stable at 0.33% for hermetic bags in contrast to 2% and 8% for metallic bins and gunny bags respectively. At end of storage, seed viability was found to be higher (88%) for hermetic bags compared to 82% and 73% in metallic bins and gunny bags respectively (Figure 4) [78,79].

Baoua et al. [48] conducted a comprehensive study to investigate the effectiveness of PICS bags (50 kg capacity) for maize storage at eleven localities in Burkina Faso, Ghana, and Benin. Insect infestation in maize during storage varied from nil to highly infested. After about 196 days of storage, the PICS bags were able to maintain 100 seed grain weight, seed viability, and seed germination along with 95%–100% insect mortality at all localities. Moisture content was also observed to be unchanged during storage for most of the PICS bags. Although aflatoxin levels were observed in maize stored in both the PICS and woven bags, the level of contamination was lower in the PICs bags. Similar effectiveness of the PICS bags was observed during storage of Bambara groundnut [80], maize [81], mung bean and pigeonpeas [67], pigeonpeas [82] (Table 4). Mutungi et al. [67] investigated the effectiveness of the PICs bags for mung beans and pigeonpeas. Naturally and artificially infested grains were stored in the PICS bags and woven bags for 6 months. For both mung beans and pigeonpeas, the oxygen levels were reduced and carbon dioxide levels rose rapidly within the two months of storage in the PICS bags. For the initial two months, the change was found to be higher in highly infested grains compared to naturally infested grains, however, at the end of storage the average change was almost the same. Insect damage and weight loss for grains remained unchanged in the PICS bags, whereas, there was 24.2–27.5 times and 21.7–43.7 times more weight loss after 6 months of storage in the woven polypropylene bags for mung beans and pigeonpeas respectively. Treatment of grains with Actellic Super dust before storage in the woven bags did not help in reducing damage, and weight loss at the end of storage increased by factors of 20.8 and 22.5 for mung beans and pigeonpeas respectively.

**Figure 5:**
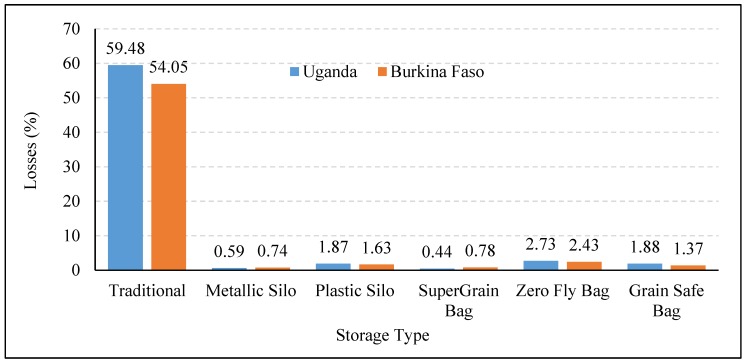
Losses in maize grain after 90 days of storage in various storage structures (data extracted from Figure 1 and Figure 2 of Costa 2014 [31]).

Figure 5 illustrates the effectiveness of various storage options compared to traditional polypropylene bags in reducing the losses of maize grains after 90 days of storage [31]. It can be clearly observed that losses in all new storage techniques were significantly lower than those in the traditional storage, with the minimum being in the case of metallic silos. A nationwide study on postharvest losses of rice in China reported 7%–13% grain losses at the rural household storage facilities, compared to only 0.2% losses at the national reserve level using scientific storage structures [64].

Metal silos have been found to be effective in several other studies. However, their initial high cost is a major obstacle for their adoption by smallholders. Community level silos might be an economic alternative, as the cost per unit of grains decreases with increases in the size of silos. The maintenance cost is very low in the case of silos, which can compensate for the high initial cost to some extent. Kimenju and Hugo [49] conducted an economic analysis of using advanced storage structures, and reported that the economic gain (extra income by avoiding losses) using a metal silo compared to polypropylene bags could be up to USD 100 per ton of grains after 12 months of storage. However, farmers have to spend an extra USD 171 (1.8-ton capacity) to USD 316 (0.36-ton capacity) as the initial cost of silos over polypropylene bags. One of the main challenges of using hermetic bags is that the grain to be stored should be thoroughly dried to avoid mold and rotting of grains. Although these bags prevent the damage from insects, they do not provide an effective barrier from rodents. Specifically, SuperGrain bags are widely used in Russia and Latin America for storage of coffee. They are also popularized in Afghanistan for wheat storage, Nepal for corn, and in Vietnam for rice conservation [86]. These bags are being used successfully in several Latin American countries to store all major grain crops without application of pesticides [74]. Hermetic storage structures developed by GrainPro Inc. are used by several commercial companies in India to store high value spices and Basmati rice [71].

Technology interventions and improved storage structures can significantly reduce store losses. However, it is important to understand that training of smallholders is equally as necessary as the technology dissemination [87]. Along with making these technologies available at a reduced price, the government agencies and organizations have to ensure the development of facilities to provide information and training about the use and maintenance of these technologies in the local language, for successful adaptation and effective use of these technologies.

## 6. Conclusions

Postharvest loss is a complex problem and its scale varies for different crops, practices, climatic conditions, and country economics. Storage losses account for the maximum fraction of all postharvest losses for cereals in developing countries, and negatively affect the farmers’ livelihoods. Most of the harvested grains are stored in the traditional storage structures, which are inadequate to avoid the insect infestation and mold growth during storage and lead to a high amount of losses. Technology interventions and improved storage structures can play a critical role in reducing postharvest losses and increasing farmers’ revenues. Hermetic storage creates an automatic modified atmosphere of high carbon dioxide concentration using the sealed waterproof bags or structures, and significantly reduces insect infestation losses. Use of properly sealed hermetic storage structures has resulted in up to a 98% reduction in storage losses, maintained seed viability, and its quality for long storage times. Using better agricultural practices and adequate storage technologies can significantly reduce the losses and help in strengthening food security, and poverty alleviation, increasing returns of smallholder farmers. 

## Figures and Tables

**Table 1:** Maturity moisture content of various crops (Source: De Lucia and Assennato [24]).

Crop	Maturity Moisture Content	Crop	Maturity Moisture Content
Paddy	22–28	Beans	30–40
Maize	23–28	Groundnut	30–35
Sorghum	20–25	Sunflower	9–10

**Table 2:** Postharvest losses of wheat from various studies in different countries.

Country	Year	Losses (%)	Comments	Reference
Bangladesh	2010	3.62	- Maximum losses during storage	[29]
India	2013	1.84	- Maximum losses during harvesting - Punjab (study locality) is a developed state, so has better storage practices	[22]
	2013	2.74	- Study conducted in Uttar Pradesh - Maximum losses during harvesting (58.4% of the total)	[37]
	2004	4.32	- Study conducted in Karnataka - Maximum losses (0.95%) during storage at field level	[33]
	2012	4.32	- About 75% losses at the farm level - Maximum losses during storage at field level (28.9% of the total loss)	[38]
	2012	8.61	- Study conducted in Madhya Pardesh - Maximum losses during storage (56% of the total losses)	[39]
	2013	7.22	- Study conducted in West Bengal - Maximum losses during storage (54% of the total)	[37]
	2013	11.71	- Study conducted in Assam - Maximum losses during threshing (28.3%) and transportation (25.2% of the total)	[37]
Peru	2012	15–25	-	[40]
Sub-Saharan Africa	2013	15.2	-	[41]

**Table 3:** Postharvest losses of maize from various studies in different countries.

Country	Year	Losses (%)	Comments	Reference
Bangladesh	2010	4.07	- Maximum losses during storage (60.4% of the total)	[29]
Ecuador	2012	10–30	- Major losses due to insect infestation during storage	[40]
Guatemala	2012	50	-	[40]
Malawai	2010	1.4	- These losses are only from farm level activities	[42]
Panama	2012	20	- Major losses at the small-scale level farm due to a lack of adequate technology	[40]
Peru	2012	15–25	-	[40]
Sub-Saharan Africa	2013	17.8	-	[41]
Tanzania	2008	4.4	- These losses are only from farm level activities	[42]
	2010	2.9		
Uganda	2009	5.9	- These losses are only from farm level activities	[42]

**Table 4:** List of the effective use of hermetic bags for various crops in developing countries

Type of Storage	Crop	Country	Duration of Storage	Investigations	Findings	Reference
SuperGrain Bags	Maize	Kenya	6 months	Evaluated performance of hermetic storage (metal silos and super grain bags) and polypropylene bags to control infestation of pests.	Metal silo was the most effective option in controlling pest infestation. Metal silo was equally effective in controlling pest infestation even without any insecticide use. Supergrain bags were effective in controlling the infestation, however, the insect mortality was not complete. Bags were perforated by a larger grain borer.	[66]
	Maize	Benin	150 days	Compared performance of hermetic bags and woven polypropylene bags for storage of maize infested with *Prostephanus truncatus* (Horn) and *Sitophilus zeamaiswas* (Motschulsky).	Moisture levels remained unchanged in hermetic bags. Growth of insects (*Prostephanus truncatus* and *Sitophilus zeamaiswas*) was significantly less in hermetic bags. There were **0.5%–6% losses** at end of storage compared to **19.2%–27.1%** losses in woven bags.	[83]
	Rice	Bangladesh	4 months	Compared performance of hermetic bags and traditional structures for storage of rice.	Moisture content of grains remained unchanged in hermetic bags. A total of **97%** seed germination in hermetic bags vs. **95%** in traditional storage. A total of **1%** damaged grains in hermetic bags in contrary to **6%** in traditional storage.	[84]
PICS bags	Mung bean, pigeonpea	Kenya	6 months	Evaluated performance of hermetic bags for naturally and artificially infested (*Callosobruchus maculatus* (F.)) grains.	One hundred grain weight, infestation, and grain damage remain unchanged in hermetic bags. There was **60.3 to 76.9%** damage in mung beans and **75.8%–95.7%** grain damage for pigeonpeas stored in woven polypropylene.	[67]
	Maize	Benin, Burkina Faso and Ghana	6.5 months	Evaluated performance of hermetic bags for preserving maize quality during storage.	There was **95%–100%** insect mortality in hermetic bags. PICS bags maintained the seed viability and germination.	[48]
	Maize	Kenya	6 months	Evaluated performance of hermetic bags for naturally and artificially infested (*Prostephanus truncates*) grains.	There was **0%–2%** weight loss in PICS bags compared to **36.3%–47.7%** weight loss in woven polypropylene bags. There was a **13%–20.1%** reduction in germination for grains stored in PICS bags compared to a **54.1%–78.4%** drop for grains stored in wove bags.	[81]
	Bambara groundnut	Maradi, Niger	7 months	Evaluated performance of hermetic bags for preserving naturally infested Bambara groundnut quality during storage.	For highly infested grains, oxygen concentrations decreased significantly in hermetic bags contrary to unchanged in woven bags. Infestation level of *C. maculatus* in woven bags was **128 times higher** than that of hermetic bags. There was a **34.8%–89.3%** decrease in seed viability in woven bags, whereas, there was **no change** in grains stored in PICS bags. Abrasions were observed in inner HDPE bags.	[80]
	Pigeonpeas	India	8 months	Compared performance of hermetic bags vs. gunny bags for storage of pigeonpeas.	Germination of infested grains in gunny bags dropped to **44.5%** compared to high germination **(77%)** for grains stored for 8 months in hermetic bags.	[82]
	Groundnuts	India	4 months	Evaluated performance of hermetic bags for preserving the quality of natural and artificial infested groundnuts.	There was a **0.8%** decrease in seed weight for groundnut stored in hermetic bags compared to **7.2%** in cloth bags. Only **1.4%** weight loss for artificially infested groundnut stored in hermetic bags compared to **39.6%** in cloth bag. There was **92.3%** germination for artificially infested seeds stored in hermetic bags compared to only **10%** in the case of cloth bags.	[85]

PICS: Purdue Improved Cowpea Storage; HDPE: high density polyethylene.
